# Obituary: Axel Rethwilm (1959–2014)

**DOI:** 10.1186/s12977-014-0085-9

**Published:** 2014-09-25

**Authors:** Ben Berkhout, Jochen Bodem, Otto Erlwein, Ottmar Herchenröder, Arifa S Khan, Andrew ML Lever, Dirk Lindemann, Maxine L Linial, Martin Löchelt, Myra O McClure, Carsten Scheller, Robin A Weiss

**Affiliations:** Department of Medical Microbiology, Center for Infection and Immunity Amsterdam (CINIMA), Academic Medical Center of the University of Amsterdam, Amsterdam, Netherlands; Institut für Virologie und Immunbiologie, Universität Würzburg, Würzburg, Germany; Jefferiss Trust Laboratories, Wright-Fleming Institute, Imperial College, London, UK; Institute of Experimental Gene Therapy and Cancer Research, Rostock University Medical Center, Rostock, Germany; Laboratory of Retroviruses, Division of Viral Products, Center for Biologics Evaluation and Research, U.S. Food and Drug Administration, Bethesda, USA; Department of Medicine, University of Cambridge, Addenbrooke’s Hospital, Cambridge, UK; Institut für Virologie, Medical Faculty Carl Gustav Carus, Technische Universität Dresden, Dresden, Germany; Division of Basic Sciences, Fred Hutchinson Cancer Research Center, Seattle, USA; Research Program Infection and Cancer, German Cancer Research Center, Heidelberg, Germany; Division of Infection & Immunity, University College London, London, UK

It is with deep sadness that the retrovirus community learned of the passing of Axel Rethwilm, Professor of Virology in Würzburg, Germany, just a few days before his 55^th^ birthday. Researchers working on foamy viruses (FV) have lost a valuable, colleague, generous mentor and a good friend.

**Figure 1:**
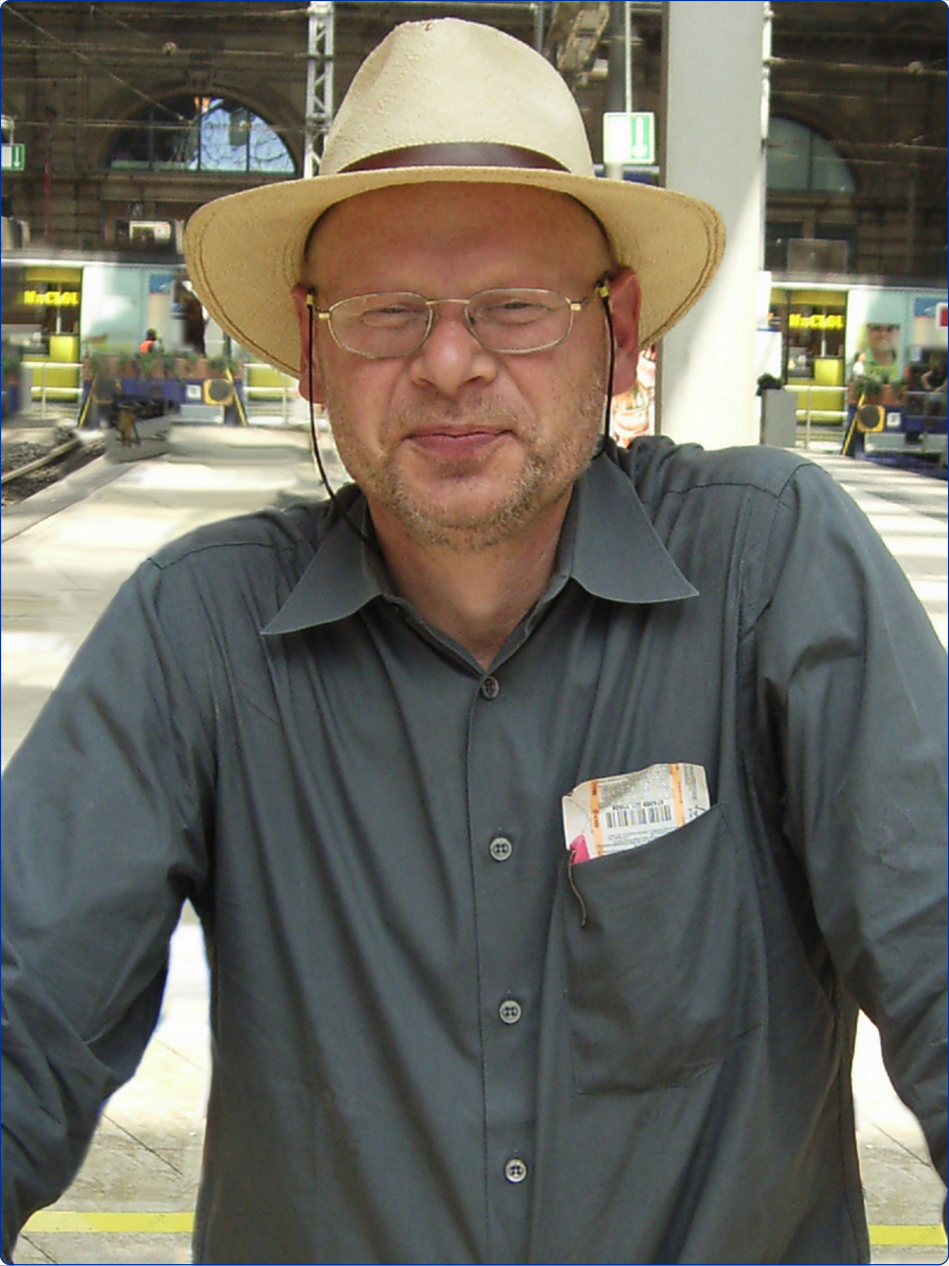
**Axel Rethwilm in 2008 at the main train station in Frankfurt am Main, Germany.** Photo provided by Ottmar Herchenröder.

Axel (Figure 1) studied Medicine in Freiburg where he also obtained his doctorate before joining the retrovirus laboratory in the Institute for Virus Research at the German Cancer Research Center in Heidelberg. Later, he moved to Würzburg to work with his mentor, Volker ter Meulen. At 38, Axel was appointed as a full Professor of Virology in Dresden and in 2003 he succeeded Volker ter Meulen to the Chair of Virology at Würzburg. With his big personality, his sharp mind, an unlimited scientific curiosity, and his refreshing openness, Axel brought to the position new and exciting ideas, and sometimes unconventional solutions.

Research active for over thirty years, Axel’s name was synonymous with FVs, his enduring passion. Since the first detection around sixty years ago of the Spumaviruses (as they were originally designated, from the Latin, *spuma*, meaning foam), more than a tenth of published FV articles have included Axel’s name as an author. His first scientific work appeared in 1983 under the guidance of the future Nobel laureate, Harald zur Hausen [1]. In the same year, HIV-1 was discovered, fuelling a retrovirology reawakening. Thereafter, Axel was frequently asked why he continued to work on a rare retrovirus that was deleterious to cell cultures, but did little else. Undeterred, Axel responded that he liked to understand all nature’s complexity.

In the first decade of Axel’s career, much of FV research was dominated by the question of which disease(s) may be caused by the so-called human foamy virus (HFV) [2]. A good number of reports associated several maladies with this isolate, and even claimed that whole sections of the human population carried the infection. For years controversy raged in the FV community over the validity of Western blots and PCR carried out on human samples. A controlled blinded evaluation of blood samples was needed and Axel organised that fresh clinical samples from thyroiditis patients were retested in three international laboratories, including his own. They proved to be FV-negative. A later larger study put an end to the dispute [3].

Subsequent to this, and in the light of emerging sequence data, the community agreed that the “viruses in search of a disease” [4] were “not so human after all”, but rather a variant of a chimpanzee foamy virus zoonotically transmitted to man as a dead-end infection [5]. Thereafter, Axel successfully campaigned to rename HFV the prototypic foamy virus (PFV). Nevertheless, humans can be infected without apparent pathology by FVs following close contact with several non-human primate species [6].

Axel was one who from the start brought molecular techniques to bear on his investigations of FVs, greatly contributing to the complete sequence of HFV. With his first HFV infectious molecular clone [7], in-depth studies on FV could lift off and Axel and others were able to discover some unique properties of FVs that distinguished them from all other retroviruses: a second promoter outside the 5’ LTR [8], translation of the polymerase gene as a separate protein from a spliced RNA and the fact that mature FV particles contain large amounts of almost full-length double-stranded DNA [9]. These features result in a unique assembly strategy [10] and the requirement of Env co-expression for particle egress [11].

Twenty years ago Axel’s group produced the first FV-based vector system [12] and for years led their further development. Today, FV vectors are showing promising results in pre-clinical testing for the treatment of inherited diseases. Alas, Axel did not live long enough to see one of his scientific goals realised: a patient treated with FV vectors in a gene therapy trial.

Axel was very engaged with the scientific community. He served on many occasions as an expert referee for various journals, including *Retrovirology*. For journal editors he was probably the first port of call for a review on any aspect of FVs and he was a conscientious, rigorous, but fair, referee. Although it was physically becoming more demanding, he enthusiastically joined his colleagues in international meetings, including the Frontiers of Retrovirology meeting that took place in Cambridge in the autumn of 2013. In February this year, he was very proud to show off the 6^th^ volume of the bible of our scientific discipline: Fields Virology 2013, with a chapter on FVs that he had written with Dirk Lindemann [13]. He never lost his enthusiasm for the latest developments in the field, and was not averse to catching up with the latest gossip. It was rare to see Axel without a smile on his face. His warmth and well-developed sense of humour, together with his unmistakable laugh, will be sorely missed at scientific meetings.

Axel suffered from a heritable, progressive motor neuron disease, which latterly limited his physical abilities. He lived bravely with increasing physical infirmity, stubbornly trying to ignore its ravishing effects for as long as possible. By pointing out that the gene responsible for his impairment was dispensable in mice, he displayed his own brand of black humour. At the most recent 10^th^ International Foamy Virus Conference in Pulawy, Poland, only seven weeks before his passing, although frail, he was as the community always knew him: interested, eager, constructively critical, supportive, a textbook of foamy- and retrovirology, conceiving fresh ideas and discussing future projects. He had become interested in a novel species of FV-encoded micro RNAs recently identified by him and others [14,15] and predicted that they would become central to FV research in the coming years. Reflecting his enthusiasm for FVs, Axel was always keen to identify more FVs, for instance from bats and other species known as key players in zoonotic events.

His increasing handicap did not restrain Axel from tiring trips into the heart of Africa. He actively supported research there and in India and was the initiator of and speaker at the first German-African Graduate School where young scientists performed research on HIV, AIDS and associated infectious diseases. Axel was interested in and dedicated to teaching. He supported several PhD and MD students from Germany and Africa by giving, from his personal funds, long-term stipends and travel grants allowing students to join national and international meetings or to visits others laboratories abroad. His work on HIV in Africa highlighted more newly infected HIV-patients with resistant virus [16] than had been estimated by the WHO. It is a pity that Axel’s excursion into the field of pathogenic retroviruses came so late in his career, although he had been seduced to working on other human pathogens in the course of more clinical studies (HIV-1, parvovirus, Epstein-Barr virus, norovirus, astrovirus, adenovirus, bocavirus, chikungunya virus).

For many working under his guidance, Axel was a great mentor and a friend beyond their time in his laboratory. Current and former lab members had, in a way, been Axel’s family. We all profited from this. We shall miss him, not only as an excellent scientist, colleague and mentor, the font of all FV knowledge, but as a friend with a strong and unique personality.

In the last review that Axel wrote before he died, he ended with the words “if I can stimulate one or more researches to pick up FV biology in their research repertoire, the mission of this review is accomplished”. The review may not have been published, but the mission was accomplished in his lifetime (Figure 1).
